# Insights From TgF344-AD, a Double Transgenic Rat Model in Alzheimer’s Disease Research

**DOI:** 10.33549/physiolres.935464

**Published:** 2025-02-01

**Authors:** Athira NATARAJ, Karel BLAHNA, Karel JEZEK

**Affiliations:** 1 Biomedical Center, Faculty of Medicine in Pilsen, Charles University, Pilsen, Czech Republic

**Keywords:** Alzheimer’s disease, Transgenic AD models, TgF344-AD rats, Spatial coding

## Abstract

Alzheimer's disease (AD), a leading cause of dementia worldwide, is a multifactorial neurodegenerative disorder characterized by amyloid-beta plaques, tauopathy, neuronal loss, neuro-inflammation, brain atrophy, and cognitive deficits. AD manifests as familial early-onset (FAD) with specific gene mutations or sporadic late-onset (LOAD) caused by various genetic and environmental factors. Numerous transgenic rodent models have been developed to understand AD pathology development and progression. The TgF344-AD rat model is a double transgenic model that carries two human gene mutations: APP with the Swedish mutation and PSEN-1 with Δexon 9 mutations. This model exhibits a complete repertoire of AD pathology in an age-dependent manner. This review summarizes multidisciplinary research insights gained from studying TgF344-AD rats in the context of AD pathology. We explore neuropathological findings; electrophysiological assessments revealing disrupted synaptic transmission, reduced spatial coding, network-level dysfunctions, and altered sleep architecture; behavioral studies highlighting impaired spatial memory; alterations in excitatory-inhibitory systems; and molecular and physiological changes in TgF344-AD rats emphasizing their age-related effects. Additionally, the impact of various interventions studied in the model is compiled, underscoring their role in bridging gaps in understanding AD pathogenesis. The TgF344-AD rat model offers significant potential in identifying biomarkers for early detection and therapeutic interventions, providing a robust platform for advancing translational AD research.

## Introduction

Alzheimer’s Disease (AD) is a progressive neurodegenerative disorder and is the leading cause of dementia [1–3]. The estimated worldwide number of individuals with AD dementia, mild cognitive impairment (MCI), and preclinical AD stood at 32 million, 69 million, and 315 million, respectively. Collectively, these counts represent 22 % of the global 50+ aged population, and with the rapidly increasing global aging population, the incidence of AD is expected to triple by 2050 [4,5]. The AD continuum has three main phases: preclinical AD, involving prolonged Aβ buildup; MCI, encompassing early-stage pathology; and Alzheimer's dementia, representing severe cognitive and functional impairment with notable brain abnormalities. AD is characterized by various pathological features, including amyloid-beta plaques, tauopathy, neuronal loss, neuroinflammation, brain volume reduction mainly in regions of cerebral cortex and hippocampus, enlargement of ventricles, decreased glucose metabolism, and oxidative stress [2,6–12]. The primary brain regions affected in AD include the medial temporal lobe and associative neocortical structures. The condition is accompanied by significant cognitive and memory deficits, impacting recent facts, spatial orientation, attention, and executive functions. Standard diagnostic methods for AD include measuring beta-amyloid and tau levels in cerebrospinal fluid and PET scanning to detect the presence of amyloid plaques and neurofibrillary tangles (NFT).

AD exists in two primary forms: Familial early-onset AD (FAD), a less frequent type characterized by inherited gene mutations such as Amyloid precursor protein (APP), Presenilin-1 (PSEN-1), Presenilin-2 (PSEN-2), and microtubule-associated protein tau (MAPT). These mutated genes also significantly increase the risk of late-onset AD (LOAD) [13]. The second form is sporadic LOAD, which has a multifactorial etiology and typically occurs in older age. The gene mutation with the highest risk factor for developing LOAD is associated with the apolipoprotein E variant ɛ4 (APOEɛ4) and triggering receptor expressed on myeloid cells 2 (TREM2) [14,15]. Besides the age and genetic predisposition, other risk factors contributing to the development of AD include vascular diseases, infections, diabetes, lifestyle and environmental factors. Notably, two prominent hypotheses shed light on the disease's underlying mechanisms. The cholinergic hypothesis, which suggests impaired acetylcholine neurotransmission mainly reflects cognitive and neuronal dysfunction of AD [16]. In contrast, the amyloid hypothesis imparts the degradation of amyloid-β and explains the formation of amyloid plaques, neurotoxicity induction, tau pathology, and subsequent neurodegeneration [17].

Despite the etiology of sporadic AD is largely unknown, animal genetic models are substantial in attempts to map and understand the development and progression of AD pathology. Numerous transgenic rodent AD models have been developed over time, with the first transgenic mouse model in 1995 [18]. Transgenic rats, in particular, offer advantages over mice due to their closer resemblance to humans in physiological, morphological, and genetic characteristics. Tg6590, UKUR25, TgF344-AD, and McGill-R-Thy1-APP are among the well-known examples of transgenic rat models in AD research [11,19]. While all AD rat models prominently express amyloid plaques, only a few, like, UKUR25, APP+PS1, Psen1^LF^, TgF344-AD rats exhibit both amyloid plaques and NFTs. TgF344-AD rats developed on a Fisher344 background co-express human APP and human PSEN-1 mutations [20]. TgF344-AD rats exhibit a complete repertoire of AD pathological features in an age-dependent manner.

The cumulative knowledge of the pathogenesis of AD from transgenic models is essential for establishing correlations with human AD to enhance future studies in translational AD research. Characterized by age-dependent cerebral amyloidosis preceding tauopathy, neuronal loss, gliosis, cognitive and behavioral disturbances, TgF344-AD rats provide crucial insights into the various stages of AD pathology. In this review, we discuss the progress made in electrophysiological, behavioral, molecular, and structural studies conducted on the TgF344-AD rat model, underscoring its importance in identifying potential biomarkers for early, preclinical detection of AD and gaining insights into the disease-modifying therapies. Our review emphasizes the unique contributions of the TgF344-AD rat model in filling existing gaps and advancing our understanding of AD.

## Prominent transgenic rat models in AD research

Various transgenic rats expressing human genes associated with FAD, including mutated APP and PS1, have been developed since the early 2000s [21]. These models display heterogeneity in their phenotypes based on the variations in neuronal promoters, the transgenes expressed, and genetic backgrounds. However, the majority of these transgenic rats exhibit the classical AD pathological hallmarks, such as amyloid pathology with mature plaques and cognitive impairment. Here, we briefly describe three conventional and most frequently used rat models, highlighting their AD-related.

### Tg6570

The Tg6590 rat model is characterized by a human APP containing the Swedish mutation under the regulation of the ubiquitin promoter in Sprague-Dawley rats [22]. The cortex, hippocampus, and cerebellum exhibit the highest expression levels. Upon reaching 15 months, animals show heightened tau phosphorylation and extracellular Aβ staining. Aβ accumulation is predominantly localized within cerebrovascular blood vessels, with occasional diffuse plaques. At 9 months, the Tg6590 rats exhibited learning and memory deficits in the Morris water maze and altered spontaneous behavior measured in open-field tests [23].

### UKUR25

UKUR25 rats express human APP with the Swedish and Indiana mutations and mutated PS1 using Wistar rats as background strains [24]. Both the transgenes are driven by platelet-derived growth factor (PDGF) promoter. The main pathological feature observed is the intracellular accumulation of Aβ in neurons within the hippocampus and cortex, with no extracellular amyloid observed up to 24 months of age. The transgenics exhibit a slight deficit in acquisition learning by the time they reach 16 months of age. In addition, the model exhibited differences in the protein profiles, including an increase in the phosphorylated form of the extracellular-regulated kinase (ERK) 2 and a decrease in the phosphorylation of the CREB kinase, suggesting potential modifications in ERK2 signaling and the tau-phosphorylation cascade [25].

### McGill-R-Thy1-APP

The McGill-R-Thy1-APP rat model is developed by introducing human APP (hAPP) with the Swedish and Indiana mutations, under the control of the murine Thy1.2 promoter, into Wistar rats [26]. In this model, the single transgene selectively expresses human APP in brain areas relevant to AD without affecting the cerebellum or peripheral tissues. From postnatal day 7 onwards, the animals exhibit intracellular Aβ inclusions across various cortical regions and the hippocampus, including CA1, CA2, CA3, and the dentate gyrus. Extracellular amyloid plaques, glial activation, and dystrophic neurites appear at 6–9 months of age. Cognitive assessments, such as the Morris water maze (MWM) and open field test, reveal an early memory impairment. Homozygous rats display altered spatial function in the MWM task as early as 3 months of age, preceding the presence of CNS plaques and this spatial impairment becomes more pronounced in older animals (13 months). At 6–12 months, the transgenic rats exhibit less activity than their wild-type counterparts in open-field tasks [27]. Other behavioral studies indicate problems with balance and gait coordination, heightened anxiety, and severely impaired spatial cognition in McGill-R-Thy1-APP rats aged 4–7 months [28].

## TgF344-AD rat, a double transgenic rat model

In 2013, Cohen *et al*. introduced the TgF344-AD rat model, characterized by the co-expression of two human gene mutations implicated in AD: hAPP with the Swedish mutation and human PSEN-1 (hPSEN-1) with Δ exon 9 mutations under the control of the murine PrP promoter [20]. Despite minimal genetic modifications, TgF344-AD rats exhibit 2.6 times higher expression of hAPP and 6.2 times higher expression of hPSEN-1 than endogenous rat proteins, inducing a spectrum of AD-related pathological features in an age-dependent manner.

## Neuropathological characterization of TgF344-AD rats

The neuropathological profile of TgF344-AD rats reveals a progressive and age-dependent pattern of pathological changes resembling that of AD in humans. The intraneuronal accumulation of Aβ1–42 and soluble Aβ1–40 is observed as early as in 6 months of age, indicating an early onset of amyloid pathology in this model. Amyloid accumulation increases significantly with age, reaching 24- and 82-fold in key brain regions such as the cingulate cortex and hippocampus at 16 and 26 months, respectively, compared to 6 months [20]. Another key pathophysiology of AD, the tau pathology which is characterized by NFT, also follows an age-dependent trajectory. Increased tau phosphorylation observed at 6 months precedes amyloid plaque accumulation in TgF344-AD rats [20]. Moreover, hyperphosphorylated tau accumulation is observed in brain regions such as locus coeruleus, preceding its occurrence in areas of entorhinal cortex and hippocampus, as highlighted in a study with rats aged 6 and 16 months of age [29]. Glial cell activation, including reactive gliosis involving microglia and astrocytes, is elevated as early as in 6 months, occurring in parallel with amyloid and tau pathology [20]. The heightened glial response is accompanied by phagocytosis of neuronal debris, reflecting ongoing neuroinflammation in the model. In addition, elevated neuroinflammatory markers and reduced anti-inflammatory marker TREM2 (triggering receptor expressed on myeloid cells 2 which is essential for microglial progression) are also observed in 18–21 months old TgF344-AD rats [30]. The model's progressive nature of neurodegeneration is also evident by neuronal loss in the entorhinal cortex and hippocampus with its first signs in age of 9 months [31] and it further develops in more significant decrease in cell counts as described in hippocampus at 16 and 26 months [20].

## Cognitive and behavioral changes

A progression of cognitive impairment in TgF344-AD rats from early adulthood to senescence has been described across numerous studies. Depending on the memory type assessed, the deficit manifested at different age levels. An important intersection between the typical clinical symptoms of cognitive disorders in AD patients and the available testing options for laboratory animals is the quantification of spatial navigation abilities. Spatial navigation and memory weren’t reported to show convincing impairment before 4–6 months but start to decline progressively thereafter. Both male and female rats exhibit spatial orientation deficits from 4 months of age that was repeatedly confirmed in higher age levels, depending on the spatial memory paradigm engaged [32–35]. Remarkably, in some studies females and males exhibited different spatial impairment profiles. Female rats tended to perform comparatively better in spatial memory tests than their male counterparts, although deficits were still evident compared to controls [49,36]. Females outperformed males in spatial learning and memory as assessed by the active place avoidance task, transgenic females exceeded transgenic males in both early acquisition and in the maximum time to avoid the shock zone after training [36]. Srivastava *et al*. showed an earlier onset of spatial memory impairment measured by active place avoidance test in male rats (at 9 and 12 months) compared to females (only at 12 months) [37]. Cognitive deficits in 15-month-old rats were further evident by impaired performance in the open field and Barnes tests and in difficulties during reversal learning in the MWM [20]. Interestingly, the TgF344-AD rats exhibited deficits in spatial reference memory and learning as early as 4 months and at 9–10 months. However, they showed intact spatial working memory at 9–10 months and cognitive resilience in executive function at 12 months [31,38,39]. Altogether, findings from a comprehensive range of behavioral tests, such as open field, Barnes maze, MWM, T-maze reference test, and Y-maze test highlight the progressive nature of cognitive decline in TgF344-AD rats, offering a valuable understanding of the development of cognitive impairment in AD. The above-mentioned behavioral tests are detailed in Table 1.

Increased anxiety often accompanies cognitive impairment in AD symptomatology. Majority of studies measuring the anxiety level showed its increase in TgF344-AD rats across sexes, however the results eventually contradict each other in details such as the age onset or sex difference. The early-onset anxiety symptoms [32] were observed in both male and female TgF344-AD rats at 4–6 months in one study. However, another study reported an effect in open field at age 6 and 12 months only in female transgenic rats, while the males did not differ from their controls [40]. Moreover, female AD animals compared to female controls and male AD rats, displayed higher anxiety-like scores at 9 months while the memory performance was not impaired [37]. Interestingly, non-stressed TgF344-AD rats aged 6–7.5 months exhibit increased anxiety-like behavior, and the acclimatization to stress has not further enhanced anxiety or fear-like behavior [41].

## Neuronal dysfunction in TgF344-AD rats

### Abnormalities in hippocampal spatial coding

More than 60 % of AD patients exhibit spatial disorientation and wandering behavior as a critical hallmark of the disease pathology [42]. Consequently, studies focusing on spatial tasks for rodents to reveal mechanisms of spatial cognition and memory impairment hold a prominent role in AD research pathology [33,43]. Hippocampal-entorhinal system is a core processing circuit for navigation in space in mammals as well for episodic memory in humans. Population of hippocampal place cells form cognitive maps or spatial representations, that code animal’s own position in known space, as well as position of other subjects including their social identity [44]. Hippocampal representation of space is considered as substrate of spatial memory. While it is uncertain in laboratory rodents to what degree/if at all it represents as well episodic memory, it expresses at least a context-independent reactivation of stored spatial patterns [45–47], a feature that is a necessary prerequisite for recalling episodes. The input information is streamed from a variety of cell types located in its projection areas, mainly from medial and lateral entorhinal cortex [48]. Abnormalities in functionality of hippocampal-entorhinal system accompanied by deficits in spatial navigation and memory have been reported in different transgenic AD models [49,50], including TgF344-AD rats. Galloway *et al*. showed a reduction in the spatial fidelity of hippocampal place cells in the CA3 and CA2 regions of 12–20-month-old TgF344-AD rats [51]. Another study focusing on 18–20-month-old TgF344-AD rats reported dysfunctional spatial tuning over repeated exposure to an open field arena, evidenced by lower spatial information content, stability, and in-field firing rates compared to their control counterparts [52]. In addition, the CA1 place cell population of younger animals (9–12 months) showed an impairment in spatial information encoding towards novel experience. This was indicated by increased place field size, decreased spatial information index, and less directional specificity in TgF344-AD rats compared to controls after an exposure to novel environment [53].

### Altered synaptic transmission

Apart changes on neuronal level, several studies showed a direct correlation of amyloid accumulation with synaptic loss and dysfunction in AD [54]. The age-related changes in synaptic transmission of TgF344-AD rats are evident with a decreased basal synaptic transmission at medial perforant path-dentate granule cell (MPP-DGC) prior to CA3-CA1 synapses, and with NMDA-dependent long-term potentiation (LTP) increase at MPP-DGC synapses at six months of age [55]. The decreased basal synaptic transmission at MPP-DGC synapses occurs without changes in dendritic spine density; however, the hippocampal parvalbumin circuit exhibits increased dendritic length and complexity at 9 and 12 months before undergoing atrophy at 15 months [31]. Additionally, degeneration of locus coeruleus-norepinephrine axons in the hippocampus coincides with increased LTP magnitude at MPP-DGC synapses in 6–9 months old TgF344-AD rats [56]. Electrophysiological analysis of 6-month-old female TgF344-AD rats revealed significant changes in passive and active membrane properties, increasing DGC excitability [57]. The basal amygdaloid nucleus (BA) synaptic dysfunction was also reported in a study examining the fear extinction memory in TgF344-AD rats, showing an impaired long-term extinction memory, hyperexcitable BA, and impaired LTP regardless of age [58].

### Molecular level alterations

The synaptic composition in TgF344-AD rats exhibited variation, characterized by reduced levels of the presynaptic marker synaptophysin, decreased density of serotonin receptors at 18 months, along with higher levels of prostaglandin D2 receptors in 11-month-old transgenics, altogether suggesting vulnerability to synapse loss in AD pathology [59–61]. The other molecular and physiological alterations observed in TgF344-AD rats consisted of changes in various protein expressions associated with glial activation, receptor composition, etc. TgF344-AD rats displayed a temporal evolution of glial involvement, showing heightened translocator protein expression in astrocytes at 12 months and in microglia at 24 months [62]. The altered protein expressions in the model included an upregulated ATP-binding cassette and solute carrier transporters in 9–10-month-old AD rats, alterations in CSF apolipoprotein species, reduction in esterified oxylipins in 10-month-old rats, and intact nuclear expression of the Repressor Element-1 Silencing Transcription factor, a regulator of neurogenesis from 6 to 18 months (unlike in controls where the transcription factor expression increases with age) [59,63–65]. Blood-brain barrier (BBB) dysfunction emerges between 13 and 18 months, evidenced by increased water permeability and elevated brain uptake of fluorodeoxyglucose in TgF344-AD rats compared to controls, pointing to an accelerated onset of BBB dysfunction in AD pathology [66,67].

TgF344-AD rats also exhibited mitochondrial dysfunction, marked by a reduced mitochondrial complex I and oxidative phosphorylation along with impaired complex II functions [68], exhibited a rapidly accelerated decline in cell respiration, resembling an age-related pattern [69]. Finally, altered neuronal metabolism and neurotransmission associated with AD progression were shown with impaired glucose clearance and reduced insulin sensitivity in female rats at 9 and 12 months [37]. Other metabolic changes included reduced cerebral and hippocampal vasoreactivity, diminished functional hyperemia, and decreased glucose uptake in 9-month-old TgF344-AD rats, as well as decreased N-acetyl aspartate and glutamate levels in TgF344-AD rats aged 9 to 18 months [70,71].

## Altered neural network in TgF344-AD rats

### Altered oscillatory network

TgF344-AD rats also exhibit network-level dysfunctions accompanied by amyloid accumulation, another characteristic of AD pathology. Compromised oscillatory interactions are indicated by lower power in the low gamma band, reduced theta-gamma phase-amplitude coupling within the cortex, and an impaired modulation index in the hippocampus and medial prefrontal cortex [72,73]. Age-dependent change in oscillations is also observed as a decrease in theta oscillation accompanied by lower power of hippocampal theta oscillation and decreased gamma power during sharp wave-ripple (SWR) events in 9–12 months old TgF344-AD rats. Impaired coherence in intercortical and hippocampal-cortical network dynamics and increased occurrence of hypersynchronous high-voltage spindles further underline hippocampal network dysfunction in AD [74]. Moreover, early alterations, such as increased soluble Aβ species in the hippocampal CA1 layer at 4–5 months, preceding amyloid plaque formation, contribute to decreased high theta oscillation power and increased SWR power [75]. SWRs are irregularly occurring large amplitude oscillations coupled to high-frequency ripple activity in the hippocampus, which plays a critical role in memory reactivation and occurs in quiet wakefulness or slow wave sleep.

In addition to neuronal network dysfunction, the electrophysiological studies also showed altered sleep patterns in TgF344-AD rats. The disrupted sleep patterns and altered wakefulness-associated brain activity are apparent in 17-month-old TgF344-AD rats. These rats exhibit increased sleep-wake transitions and a higher probability of shorter REM and NREM bouts than controls [76]. Moreover, a more uniform distribution of relative spectral power during wakefulness, reduced EEG information during wakefulness, and increased information during NREM sleep indicate impaired sleep architecture in AD [76].

### Altered inhibitory and excitatory system

The changes in the excitatory system and GABAergic signaling mediated inhibitory system emphasize another complex aspect of the AD progression in TgF344-AD rats. At 9 months, while the expression of GABA receptor subunits remained unchanged, the glutamate decarboxylase/GAD (GAD catalyze the conversion of glutamate to GABA) – positive cells are less expressed in the CA1 region of the hippocampus of TgF344-AD rats, indicating impaired GABAergic neurotransmission [73]. Additionally, inhibitory receptor-mediated modulation of ripple dynamics in the CA1 region is compromised in 9-month-old TgF344-AD rats, and by 12 months hippocampal neurons exhibited compensatory mechanisms with increased expression of GABAergic interneuron marker GAD67 [31,77]. However, by 15 months, significant loss of both excitatory and inhibitory neurons occurred, indicating progressive neuropathology. GABAergic interneurons are well known for their role in regulating the balance and dynamics of pyramidal cell ensembles and the types of GABAergic interneurons include somatostatin and parvalbumin interneurons. The somatostatin interneurons displayed early tau effects starting at 9 months, while parvalbumin interneurons remained resilient until 15 months, suggesting differential vulnerability among GABAergic interneuron subtypes during AD progression [31].

Regarding the excitatory system, TgF344-AD rats displayed a simultaneous decline in locus coeruleus-noradrenergic axons and an increase in β-adrenergic receptor activity at MPP-DGC synapses at 6–9 months. The heightened β-adrenergic receptor function led to increased LTP, indicative of a compensatory mechanism to preserve cognitive function during AD development [56].

## Multifaceted system impairments

Other functional impairments observed in TgF344-AD rats include a range of deficits, such as vision, neurovascular, auditory, and olfactory impairments. These impairments are summarized in Table 2 [78–83].

## Age dependent functional alterations and other factors influencing AD progression

Functional alterations in TgF344-AD rats are characterized by age-specific changes in network dynamics, elucidating the temporal dynamics of AD pathology.

Pre-Plaque and Early Plaque Stages (4–6 months): During the pre-plaque stages, altered cortical excitatory/inhibitory balance, decreased basal forebrain (BFB) quasi-periodic pattern (QPP) of neural activity, and compensatory mechanisms in response to synaptic changes are observed [84]. Resting-state functional MRI (rsfMRI) and co-activation pattern (CAP) analysis reveal reduced co-activation of hub regions, particularly in default mode-like networks, providing insights into early AD-related functional alterations [85].

Early Changes (5–6 months): At 5 months, global and regional structural network disruptions indicate early brain connectivity disturbances [86]. Functional connectivity decreases slightly, particularly in somatosensory, sensorimotor, and anterior default mode networks [87]. Furthermore, decreased BFB QPP neuronal activity and increased BFB astrocytes suggest synaptic alterations preceding plaque formation [84].

Mid-Stage Changes (6–10 months): Between 6 and 10 months, female TgF344-AD rats experience a significant decrease in functional connectivity, progressing from mild to severe and widespread hypoconnectivity, and these changes precede microstructural alterations [88]. PET scans at 6 months reveal decreased cerebral protein synthesis rates in the globus pallidus, reflecting neurodegenerative processes in mid-stage AD [89].

Late-Stage Changes (10–18 months): By 10 months, hippocampal neurochemical alterations become apparent, including increased total choline and lactate and decreased total creatine and taurine levels [39]. Structural changes such as dentate gyrus hypertrophy and atrophy in various brain regions are observed by 16 months, indicating advanced AD pathology [39]. PET scans reveal hemispherically asymmetric neurodegeneration, accompanied by increased microglial activity and altered glucose metabolism, further confirming late-stage AD progression [35].

## Other factors influencing AD progression in TgF344-AD rats

**Fig. 1:**
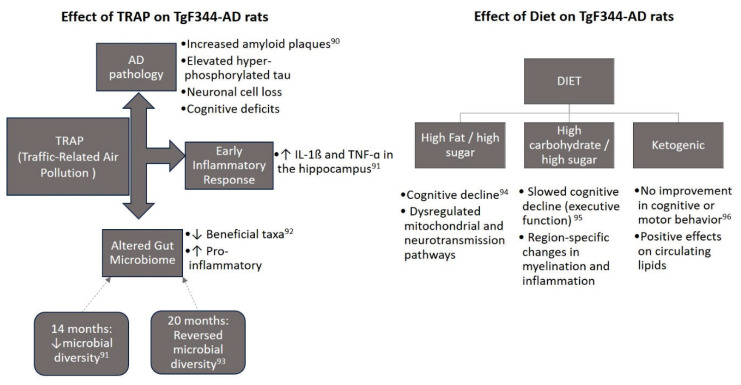
Effects of TRAP and Diet on TgF344-AD rats.

Various external factors such as pollution, microbiome composition, and dietary interventions have been explored to understand their influence on AD progression in TgF344-AD rats. The effects of Traffic-Related Air Pollution (TRAP) and Diet on TgF344-AD rats are shown in the Figure 1 [90–96].

## Unconventional AD-associated markers

In addition to more or less [97] conventional evaluation of protein expressions, several studies explored other AD associated markers in TgF344-AD rats. Piezo1, a non-selective cation channel which plays a crucial role in mechanotransduction, exhibited increased expression in neurons across different brain regions in 12-month-old rats, including the optic tract, corpus callosum, and cerebellum [98]. Reactive astrocytes surrounding amyloid plaques also showed upregulated Piezo1 expression. Additionally, aging and amyloid plaque pathology further amplified Piezo1 expression in cortical areas, suggesting its involvement in AD-related neurodegeneration.

Another underexplored marker associated with AD is capillary endothelial inward rectifier potassium 2 (Kir2.1). The well-known role of Kir2.1 includes regulating heart rhythm, muscle contraction, bone development, and other functions. Kir2.1 mutations result in cardiomyopathies, neurological disorders, and metabolic disorders. Evaluation of Kir2.1 expression in endothelial cells of TgF344-AD rats aged 3 to 14 months showed early elevation of Aβ, impaired hyperemic responses, and reduced Kir2.1 expression observed at 6 months suggesting a potential role of Kir2.1 dysfunction in cerebrovascular alterations linked to AD progression [99].

Other promising markers for detecting AD-related protein aggregates include new super luminescent conjugated polyelectrolyte molecules, oligo-p-phenylene ethynylenes (OPEs), OPE12− and OPE24+. With minimal non-specific staining, these molecules effectively stained tau-paired helical filaments and amyloid-β plaques in TgF344-AD rats and human brain sections [100]. Their superior performance both in terms of sensitivity and specificity compared to traditional markers like Thioflavin T, suggests their potential in detection of AD pathology.

## Substance/treatment induced alterations in TgF344-AD rats

Various substances, treatments, and exercises have shown promise in alleviating AD pathology in TgF344-AD rats, with effects often varying with age. The effect of prominent interventions studied in TgF344-rats are detailed in the Table 3 [101–108].

## Limitation of the model

Although the TgF344-AD rat model significantly contributes to AD research by offering insights into AD pathology through combined prisms of amyloid beta and hyperphosphorylated Tau in an age-dependent manner, it also poses few limitations. The main and obvious limit is the TgF344-AD validity in modelling human Alzheimer’s disease. The vast majority of Alzheimer's disease (AD) cases are sporadic, lacking a strong genetic predisposition and originating from largely idiopathic causes. This form of AD, which constitutes the primary burden on the population, is not encompassed by any genetic model, including the one discussed here. The AD pathophysiology is still largely unknown, and while the Amyloid Cascade Hypothesis [109] seemed promising at the times of its formulation, today there is spreading skepticism about the causative role of Aβ in AD [110]. While the rat biological background is closer to humans than the mice-one, interactions between human transgenes and rat physiology produce a unique phenotype that deviates from the desired human clinical picture. One of the striking examples would be the absence of precognitive symptoms that appear before the expression of learning and memory impairments. Among numerous one can mention neuropsychiatric symptoms (e.g. increased anxiety) and sleep/wake cycle disruptions. These signs in humans often precede cognitive symptoms by 10–20 years and largely constitute the early stages of the disease. In general, mice nor rat models do not show such dynamics. Moreover, specifically the anxiety level is rather commonly tested behavioral parameter that returns a high variability across considerable number of studies. Increased anxiety is present only at certain ages and in specific sex groups but absent at others, complicating straightforward interpretation of results [32,34,40].

A practical challenge includes breeding difficulties, as female TgF344-AD rats are less likely to become pregnant after four months of age, and their average litter size is smaller compared to wild-type rats. These issues complicate breeding and experimental timelines. Consequently, small sample sizes in some studies reduce statistical power, restricting the ability to draw definitive conclusions about cognitive changes over time. Overcoming these limitations can enhance the reliability and generalizability of findings, thereby contributing to a deeper understanding of Alzheimer's disease.

## Concluding remarks

Overall, TgF344-AD, the double transgenic rat model exhibits an age-dependent AD pathology, including amyloid-beta plaques, tau hyperphosphorylated, neuronal loss, neuroinflammation, and cognitive and behavioral deficits. Electrophysiological studies indicate impairments at both cellular and network levels. Cellular abnormalities in this model include impaired hippocampal place cell coding and altered expression of proteins involved in synaptic plasticity, glial activation, mitochondrial function, and metabolism. System-level impairments are widespread and include disruptions in brain oscillations, alterations in excitatory and inhibitory neurotransmission, changes in sleep patterns, and various sensory and neurovascular dysfunctions. External factors, such as pollution, worsen AD pathology by promoting inflammation and altering microbiome composition. However, dietary interventions have produced mixed results, highlighting the complexity of their interaction with AD progression. Additionally, various substances such as P7C3-S243 and donepezil, treatments like DBS and LD-RT, and exercise have shown improvement in AD pathology in TgF344-AD rats, with effects depending on age.

**Fig. 2:**
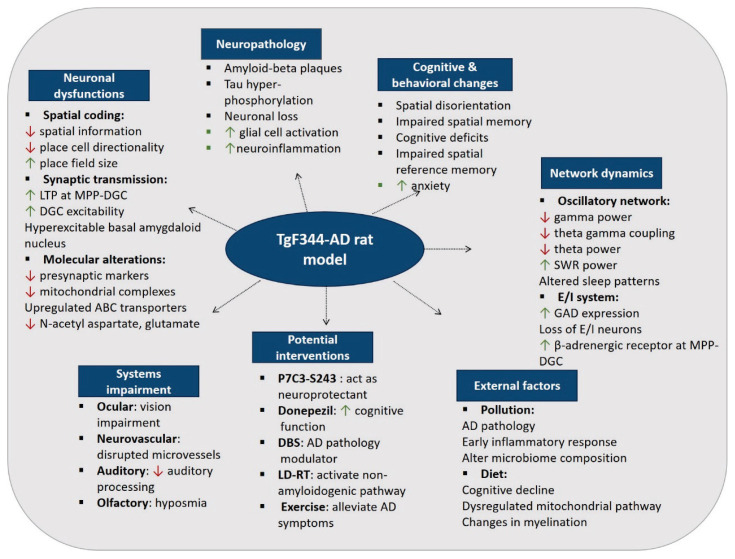
Overview of findings and interventions in the TgF344-AD rat model.

The diagram below illustrates an overview of each section, summarizing the important findings and highlighting processes, specific markers, and interventions associated with the TgF344-AD rat model (Fig. 2).

To the best of our knowledge, this review provides the first comprehensive evaluation of the TgF344-AD rat model, underscoring its potential in understanding AD pathogenesis and evaluating potential therapies. By integrating findings from multiple disciplines, this review covers a wide range of AD aspects, including phenotype, pathology, neuronal networks, cognitive and behavioral functions, functional networks, the excitatory-inhibitory system, AD-associated markers, and potential interventions. It draws on extensive experimental evidence to demonstrate that TgF344-AD rats exhibit face validity, closely mirroring human AD symptoms, including its etiology and the progression of its time-dependent pathophysiology. The review's findings significantly advance our understanding of AD and pave the way for developing more effective treatments.

## Figures and Tables

**Table 1:** Behavioral tests for assessing cognitive impairment in rodents.

*Open field*	The open field test is a common measure of exploratory and anxiety behavior in rodents, and the main outcome is based on locomotion in unfamiliar well-lit open area, along with other variables such as freezing, thigmotaxis, rearing, amount of defecation, etc.
*Barnes maze*	Barnes maze is a dry-land based spatial memory test, it consists of elevated circular platform with holes and the goal is for the animal to find the escape hole by means of visual cues provided during training sessions.
*Morris water maze*	The Morris water maze is the most popular behavioral test for spatial learning and memory, where the rodents swim from the starting point to an escape platform in a circular tank with water based on distant cues.
*T-maze*	The T-maze consists of a T-shaped apparatus with a start arm and two lateral goal arms and is an ideal task for evaluating spatial working memory. The principle is based on the exploring tendency of rodents to choose a novel arm over familiar arm and alternating the choice of goal arm across repeated trials.
*Y-maze*	Y-maze test is similar to T-maze and is used to test spatial working and reference memory in rodents, it is based on innate curiosity of animals to explore unvisited areas. However, it has three similar arms which are kept 120° to each other and exhibits a faster learning time.

**Table 2:** Functional impairments in TgF344-AD rats.

*Ocular Dysfunction*	12 months (females): Significant vision impairment [34]. 19 months: Ocular abnormalities, such as Aβ accumulation in retinal sections, decreased choroidal thickness, and inflammatory response in eyes [78]. Other findings: Functional ultrasound on the retina showed changes in neurovascular coupling [79].
*Neurovascular Dysfunction*	9 months: Amyloid plaque distribution in insular, piriform, and entorhinal cortices; tau hyperphosphorylation; increased amyloid load in cortical arterioles; decreased reactivity to hypercapnia [72]. 12 months: Amyloid-beta disrupts hippocampal microvessels and alters capillary length [80]. Other findings: Early vascular dysfunction indicated by arteriolar and venular amyloid accumulation, impaired myogenic responses [81,82].
*Auditory Dysfunction*	9–12 months: Deficits in sensory information processing, diminished auditory evoked potentials, and steady-state responses [74].
*Olfactory Dysfunction*	6–12 months: Hyposmia (reduced sense of smell) observed in males, not in females [40].
*Respiratory Control*	8–11 months: Observed no prodromal respiratory control changes related to AD; Also, no tau hyperphosphorylation, Aβ accumulation, or neuroinflammation in the brainstem [83].

**Table 3:** Effects of prominent interventions in TgF344-AD rats.

Intervention	Effect on TgF344-AD rats	Conclusion	Ref.
*P7C3-S243*	Protection against depressive-like behavior at 15 months and cognitive deficits at 24 months. No effect in amyloid plaque deposition, tau hyperphosphorylation, or glial reaction.	P7C3-S243 acts as a neuroprotective compound in AD symptom management rather than altering underlying pathology.	101
*Donepezil*	Improved hippocampal theta oscillation power and theta phase-gamma amplitude coupling in TgF344-AD rats at 6 and 12 months. Also modulated cortical spectral density and frontal-occipital phase locking.	Donepezil possibly enhances cognitive function and neural network activity across different age groups of TgF344-AD rats.	102
*Umbilical Cord Perivascular Cells*	Combinatorial treatment with umbilical cord perivascular cells and Aβ clearance rescued vascular function in TgF344-AD rats following transient hypertension.	It acts as a potential therapeutic approach to address cerebro-vascular dysfunction associated with AD.	103
*Deep Brain Stimulation (DBS*	Chronic fornix DBS reduced amyloid plaques, astrogliosis, microglial activation, and neuronal loss in 18-month-old TgF344-AD rats.	High potential role of DBS modulating AD pathology even at advanced stages.	104
*Low-Dose Brain Radiation Therapy (LD-RT)*	LD-RT reduced neuroinflammation markers and amyloid forms while increasing sAPPα levels in 9-month-old TgF344-AD rats. LD-RT daily administration improved memory performance in TgF344-AD rats aged 15 months.	LD-RT exhibits neuro-protective effect and activate non-amyloidogenic pathways.	105,106
*Exercise*	Long-term treadmill exercise starting at 2 months improved behavioral deficits and reduced amyloid-beta deposition, tau hyperphosphorylation, neuroinflammation, and oxidative stress at 12 months. Similarly, exercise in the late stages of AD pathology (starting at 18 months) alleviated memory impairment, depressive-like behavior, neuronal degeneration, and synaptic loss while improving mitochondrial function and reducing oxidative stress and neuroinflammation.	A promising role of exercise in AD intervention.	107,108
